# Examining factors influencing the adoption of smart integrated devices by the elderly in the digital era: insights from behavioral design theory

**DOI:** 10.3389/fpsyg.2025.1540201

**Published:** 2025-06-17

**Authors:** Sijie Sun, Na Qi, Haoran Li, Liang Xiao

**Affiliations:** ^1^Department of Philosophy, Autonomous University of Barcelona, Barcelona, Spain; ^2^Department of Design Graduate Schools, Sungkyunkwan University, Seoul, Republic of Korea; ^3^School of Art, RMIT University, Melbourne, VIC, Australia

**Keywords:** smart integrated devices, elderly usage behavior, behavioral design theory, digital inclusion, internet usage experience

## Abstract

**Introduction:**

Digitalization and aging are two defining characteristics of contemporary social transformation. As smart integrated devices become increasingly embedded in everyday life, understanding how elderly individuals interact with these technologies is essential for promoting digital inclusion and social integration. Although previous research has examined digital engagement among older adults, the specific behavioral and contextual factors that shape their usage of smart devices in a digital society remain underexplored. This study seeks to clarify how these factors operate and how they may be leveraged to support the well-being of the aging population.

**Methods:**

This study utilized data from the 2020 China Longitudinal Aging Social Survey (CLASS), focusing on elderly individuals living in urban areas. A combination of logistic regression analysis, Lasso regression, and robustness tests was employed to identify the key predictors of smart integrated device usage. The analysis examined a range of variables including demographic characteristics, health status, education, income, internet usage experience, and family structure. Comparisons were made across groups to assess how these factors influence usage behavior, and Lasso regression was used to identify the most robust predictors.

**Results:**

The analysis revealed that elderly individuals are more likely to use smart devices if they are male, older, married, in poorer health, more highly educated, have lower income, have fewer children, and have previous experience using the internet. Among these, internet usage experience emerged as the most significant and consistent predictor across all models, as identified by the Lasso regression. Furthermore, the purposes for which the elderly use the internet, such as communication, information, or entertainment. These patterns were found to be stable even after controlling for potential confounding variables.

**Discussion:**

The findings challenge traditional assumptions about fixed sensory or cognitive hierarchies in aging populations and instead suggest that smart device usage among the elderly is shaped by a dynamic interplay of motivational, ability-based, and environmental factors. Drawing on Behavioral Design Theory, the study interprets these patterns through three lenses: motivation (e.g., health monitoring and social interaction), ability (e.g., physical and cognitive usability of devices), and triggers (e.g., technical and emotional support from family members). Under this framework, digital engagement appears to be highly context-dependent, with adaptive resource allocation and social support playing a crucial role in determining whether and how elderly individuals use smart technologies. The results emphasize the importance of designing inclusive digital environments and policies that respond to the nuanced needs and experiences of the aging population.

## 1 Introduction

With the rapid development of information technology and the acceleration of global aging, digitalization and aging have become defining features of contemporary social transformation (Francis et al., 2019). Digital technologies have profoundly reshaped lifestyles, work practices, and social interactions, influencing nearly every aspect of daily life. At the same time, the growing elderly population has raised significant concerns about their well-being, social integration, and quality of life (Repetti and Fellay-Favre, 2024; Wang et al., 2024). Understanding how digital technology can support older adults is essential for promoting their integration into modern society.

Despite the numerous benefits brought by digitalization, older adults still face various challenges when using smart integrated devices such as smartphones, tablets, and smart home systems. Physiological factors (including declining vision and motor skills), psychological barriers (such as technophobia), and social obstacles (such as limited digital literacy) create difficulties in adopting these technologies (Backåberg et al., 2025). These barriers highlight the necessity of studying the determinants of smart device adoption among older adults and developing user-friendly solutions to bridge the digital divide (Choudrie et al., 2022).

The primary objective of this study is to examine how socioeconomic, psychological, and physiological factors influence the adoption and use of smart devices among the elderly. While existing research has primarily focused on aging-related aspects such as health, economic status, and social support, limited attention has been paid to digital behavior among older adults. For instance, a study on predictors of physical activity levels among older adults found that gender, education level, recreational activities, and health status are key factors influencing physical activity. Engaging in leisure activities such as games and visiting friends indirectly contributes to increased physical activity (Parra-Rizo et al., 2022). Understanding the environmental characteristics that enhance physical activity among older adults is crucial for promoting active and healthy aging. However, limited scholarly attention has been given to the digital behavior of older adults. This study aims to fill this gap by analyzing the mechanisms influencing digital participation, thereby expanding research in gerontology, sociology, and digital inclusion.

This study draws on multiple theoretical frameworks to analyze the adoption of smart devices among older adults. The Technology Acceptance Model (TAM) emphasizes that perceived ease of use and perceived usefulness are critical factors influencing technology adoption. However, cognitive decline and limited social support may weaken these perceptions among older adults, thereby affecting their likelihood of adopting smart devices (Guner and Acarturk, 2020). Additionally, Social Network Theory posits that social support networks play a vital role in technology adoption. Older adults often rely on children, relatives, and community members for assistance, and this social support can enhance their interest and confidence in using smart devices (Zhang et al., 2021).

The core theoretical framework of this study is Behavioral Design Theory, which suggests that technology adoption is influenced by motivation, ability, and triggers (Abdukadirov, 2016; Tussyadiah, 2017; Zacher and Froidevaux, 2021). For older adults, motivation primarily stems from health management and social connection. Smart devices offering health monitoring functions and social interaction tools can increase their willingness to adopt these technologies. Regarding ability, the design of smart devices should accommodate the physiological and cognitive characteristics of older adults, such as simplified interfaces, larger fonts, and voice control functions. Triggers refer to external factors that encourage the continued use of smart devices, such as health monitoring reminders, encouragement from family members, and digital skills training provided by governments and communities. These external supports help boost confidence in technology use and facilitate long-term adoption.

This study focuses on the urban elderly population in China, utilizing a nationally representative dataset and advanced analytical methods to investigate the factors influencing smart device adoption. Data from the 2020 China Longitudinal Aging Social Survey (CLASS) is used, employing logistic regression analysis, Lasso analysis, and robustness tests to conduct a comprehensive empirical examination of the determinants of smart device usage among older adults. The findings provide valuable insights for public policy design and the development of accessible technologies, facilitating the digital inclusion of the elderly in modern society.

## 2 Literature review and theoretical hypotheses

The Technology Acceptance Model (TAM) has been widely used to explain the adoption of new technologies. It posits that perceived ease of use and perceived usefulness are the primary determinants of an individual’s willingness to embrace technology (Davis, 1989; Guner and Acarturk, 2020). For older adults, however, cognitive decline and physical limitations can significantly hinder their perceived ease of use, making it difficult for them to navigate digital interfaces (Brydie, 2009; Charness and Boot, 2016). Additionally, social and economic factors influence their perceived usefulness, as individuals with greater financial security and digital literacy are more likely to see smart devices as beneficial (Venkatesh et al., 2012). Research suggests that interventions such as digital skills training and the implementation of user-friendly designs can mitigate these challenges, making technology more accessible for the elderly (Chen and Chan, 2014).

While TAM provides an essential framework for understanding individual decision-making in technology adoption, it does not fully account for the impact of social structures. Social Network Theory offers a complementary perspective, emphasizing the role of interpersonal relationships in shaping technology use (Marin and Wellman, 2011; Zhang et al., 2021). Many elderly individuals have limited social circles and rely on their children, extended family, and community members for technological assistance (Charness and Boot, 2016). Luo (2023) highlights the significance of intergenerational digital support, showing that younger relatives play a crucial role in helping older adults learn and use digital devices. Additionally, strong social connections can increase motivation to adopt smart technology, particularly when it facilitates meaningful interactions and access to relevant content. Studies indicate that older adults who are more socially connected are also more likely to engage with digital tools (Goddard, 2025; Tsai, 2012), suggesting that digital inclusion initiatives should focus not only on individual ability but also on strengthening social networks.

Beyond individual cognition and social influence, the design and usability of technology play a fundamental role in adoption among older adults. Behavioral Design Theory underscores the importance of motivation, ability, and triggers in driving behavioral change (Fogg and Euchner, 2019).

First, motivation is a crucial factor influencing technology adoption among older adults. Many elderly users are driven by intrinsic and extrinsic motivations (Ademi et al., 2019). Intrinsically, they seek to maintain independence, improve their quality of life, and stay cognitively engaged. Extrinsically, concerns about health management, social inclusion, and pressure from family members can encourage them to adopt digital tools (Ma et al., 2023). Studies have shown that older adults who perceive clear personal benefits—such as the ability to monitor their health through wearable devices (Liang et al., 2022) or communicate with distant family members—are more likely to embrace technology (Bisset and Lockton, 2010; Blohm and Leimeister, 2013).

Second, ability refers to an individual’s capacity to successfully use a technology, which can be influenced by physical, cognitive, and technological barriers (Nielsen et al., 2021). Physically, reduced vision, hearing, and dexterity make standard interfaces difficult to navigate. Cognitively, memory decline and unfamiliarity with digital interfaces can create additional hurdles. Technologically, older adults often struggle with complex user interfaces, frequent system updates, and a lack of prior digital experience. To enhance usability, design interventions such as simplified navigation (Heree et al., 2022), larger fonts, high-contrast visuals, haptic feedback, and voice-controlled features can significantly reduce these barriers and improve accessibility (Rachmad, 2022; Sespiani and Ernungtyas, 2022; Yekinni and Ogbuanya, 2025).

Finally, triggers serve as external stimuli that prompt older adults to engage with technology. These can be reminders, social encouragement, or necessity-driven interventions. For instance, health-related notifications—such as medication reminders or fall detection alerts—provide direct benefits that encourage sustained use. Social triggers, such as family members introducing and guiding older adults through digital platforms, also play a critical role. Matsumura et al. (2015) found that seniors who receive consistent external encouragement, whether from caregivers, peers, or healthcare professionals, are more likely to integrate digital devices into their daily routines. Additionally, external supports like structured training programs and user-friendly onboarding experiences can reinforce long-term engagement (Ge et al., 2021).

By addressing these three components—enhancing motivation through personal relevance, improving ability via inclusive design, and reinforcing usage with effective triggers—technology can be made more accessible and appealing to older adults, ultimately fostering greater adoption (Schroeder et al., 2023) and integration into their daily lives.

Demographic and socioeconomic factors further influence the adoption of smart devices among older adults. Gender differences are evident, with men demonstrating greater confidence and interest in digital activities compared to women (Venkatesh et al., 2012; Heart and Kalderon, 2013; Pelau et al., 2021). While it is commonly assumed that younger elderly individuals are more likely to adopt technology, some studies suggest that older segments of the elderly population show higher adoption rates due to the necessity of staying connected and accessing essential services (Peek et al., 2014; Gell et al., 2015; Choudrie et al., 2018). Marital status also plays a role, as married elderly individuals are more likely to use smart devices due to spousal support and shared interests (Mitzner et al., 2010; Thielke et al., 2012). Additionally, while poor health can deter technology use, it can also serve as a driver for those seeking digital health management solutions (Mitzner et al., 2010; Heart and Kalderon, 2013). Education and income levels have been found to correlate with technology adoption, with higher educational attainment linked to greater digital engagement (Friemel, 2016) and lower income levels acting as a potential barrier (Choi and DiNitto, 2013; Helsper and Reisdorf, 2017). Prior experience with the internet enhances digital confidence, reducing anxiety and improving technology use (Van Deursen and Van Dijk, 2011; Quan-Haase et al., 2018). Furthermore, the number of children influences reliance on smart devices for social interaction, as elderly individuals with fewer children often use digital tools to compensate for reduced face-to-face interaction (Cotten et al., 2013; Tsai et al., 2017; Nimrod, 2017).

Based on these insights, the study proposes the following hypotheses:

*H1:* Male elderly individuals are more likely to use smart integrated devices compared to female elderly individuals.

*H2:* Older elderly individuals are more likely to use smart integrated devices compared to younger elderly individuals.

*H3:* Married elderly individuals are more likely to use smart integrated devices compared to unmarried, widowed, or divorced elderly individuals.

*H4:* Elderly individuals with lower health status are more likely to use smart integrated devices for health monitoring.

*H5:* Elderly individuals with higher educational attainment are more likely to use smart integrated devices.

*H6:* Elderly individuals with lower income levels are more likely to use smart integrated devices.

*H7:* Elderly individuals with prior internet usage experience are more likely to use smart integrated devices.

*H8:* Elderly individuals with fewer children are more likely to use smart integrated devices for social interaction and support.

These hypotheses provide a structured approach to understanding the diverse factors influencing smart device adoption among the urban elderly population in digital era.

## 3 Data and methodology

### 3.1 Data source

This study utilizes data from the 2020 China Longitudinal Aging Social Survey (CLASS), a nationally representative dataset conducted by Renmin University of China in collaboration with various research institutions and government agencies. CLASS aims to provide high-quality, policy-relevant data on China’s aging population to inform academic research and policy development in elderly care and social welfare.

CLASS employs a multi-stage stratified probability sampling method to ensure national representation, covering a wide range of provinces and balancing urban and rural populations. The survey spans across Eastern, Central, and Western China, including key provinces such as Beijing, Shanghai, Jiangsu, Zhejiang, Guangdong (Eastern); Henan, Hubei, Hunan, Anhui (Central); and Sichuan, Chongqing, Shaanxi, Gansu (Western). This broad geographical coverage ensures that the dataset accurately reflects regional differences in aging trends, socio-economic conditions, and healthcare accessibility.

The sampling process consists of four stages:

Province-Level Selection: Ensuring nationwide representation by covering a mix of developed and developing regions.
City, County, and District-Level Selection: Including urban centers, mid-sized cities, and rural areas to capture diversity.
Community-Level Selection: Targeting residential committees and village units within selected urban and rural areas.
Household Selection: Randomly selecting one elderly individual (aged 60 and above) per household for detailed interviews.

Data collection was conducted primarily through face-to-face structured interviews, where trained surveyors collected information on a wide range of topics, including demographic characteristics, socio-economic conditions, health status, and social participation. Additionally, community-level data were gathered to assess environmental factors such as eldercare services, medical facilities, and social infrastructure. To ensure the accuracy and reliability of the dataset, CLASS employs rigorous data quality control measures, including standardized interviewer training, validation checks, and logical consistency tests. This extensive data collection process allows CLASS to serve as a gold-standard dataset for studying aging in China, widely used in both academic research and policy analysis. The longitudinal nature of the survey enables tracking of aging trends over time, making it an invaluable resource for understanding the evolving needs of elderly populations in China.

By utilizing this extensive dataset, this study aims to provide detailed, evidence-based conclusions to inform policy development and interventions that enhance digital inclusion and quality of life among China’s elderly population. Based on the World Health Organization (WHO) definition of the elderly and considering that 60 years old is commonly regarded as the retirement threshold in China, this stage marks significant changes in individuals’ social roles, lifestyles, and technology usage needs, while also posing new challenges in adapting to digital technologies. Therefore, this study selects data from individuals aged 60 and above as the research sample, allowing for a more precise analysis of the behavioral characteristics and influencing factors of elderly individuals in smart device usage, ultimately providing more targeted empirical support for improving their digital inclusion.

### 3.2 Variables

#### 3.2.1 Dependent variable

The dependent variable in this study is the use of smart integrated devices. This is measured using the CLASS 2020 survey question:

*“Do you use any of the following smart devices—smart integrated devices (*e.g.*, Baidu Xiaodu, Xiaomi Xiaoai)?”*

*Responses are coded as 1 = Yes and 0 = No, making this a binary outcome variable.*

#### 3.2.2 Independent variables

We consider multiple individual- and household-level variables:

Individual-Level Variables:

*Education Level: Measured on a 7-point scale (1 = No formal education, 7 = Bachelor’s degree and above). Higher values indicate higher education levels.*

*Internet Usage Experience: Binary* var*iable (1 = Yes, 0 = No), based on the question: “Do you use the internet (including mobile phones or other electronic devices)?”*

*Self-rated Health: Measured using the question: “How would you rate your current health status?” with five response options:*

*“Very unhealthy,” “Relatively unhealthy,” “Average,” “Relatively healthy,” and “Very healthy.”*

Due to the binary nature of the dependent variable and for ease of interpretation, responses are categorized as:

*1 = Healthy (combining “Relatively healthy,” “Average,” and “Very healthy”).*

*0 = Unhealthy (combining “Very unhealthy” and “Relatively unhealthy”).*

This consolidation simplifies analysis while preserving meaningful distinctions in health status.

*Personal Income: Measured as total annual income in the past 12 months, log-transformed for analysis to mitigate skewness.*

Household-Level Variables:

*Number of Children: Total number of biological and adopted children, reflecting family support availability.*

*Marital Status: 1 = Married with spouse, 0 = Widowed, divorced, or never married, reflecting the impact of spousal support on technology adoption.*

Descriptive statistics of the main variables are shown in Table 1.

**Table 1:** Summary statistics.

Variables	(1)	(2)	(3)	(4)	(5)
	*N*	Mean	sd	min	max
Gender	2,330	0.479	0.500	0	1
Education	2,330	3.989	1.087	1	7
Income	2,330	13,819	13,913	450	240,000
Smart_device	2,330	0.171	0.376	0	1
Age	2,330	70.84	6.763	60	97
Living_status	2,330	0.805	0.397	0	1
Health	2,330	0.522	0.500	0	1
Internet	2,330	0.549	0.498	0	1
Child	2,330	1.775	1.025	0	8
Province	2,330	10.384	8.233	1	26

### 3.3 Methodology

Due to the binary nature of the dependent variable (use or non-use of smart devices), this study employs logistic regression analysis, a widely used statistical method for examining the factors influencing binary outcomes. The independent variables include gender, age, living status, health status, education level, income level, internet usage experience, and the number of children. Logistic regression is used to quantify the impact of each factor on the likelihood of smart device adoption among the elderly:


$$\begin{array}{l} \text{logit}(P(\text{Using Smart Integrated Devices}=1))\\ =\beta_{0}+\beta_{1}\text{gender}+\beta_{2}\mathrm{age}+\beta_{3}\text{livin}g_{\text{status}}+\beta_{4}\text{health}\\ +\beta_{5}\text{education}+\beta_{6}\text{income}+\beta_{7}\text{child}+\beta_{8}\text{internet} \end{array}$$

Among them, $\beta_{1}-\beta_{8}$ are the regression coefficients of their respective variables. By calculating odds ratios $e^{\beta_{1}-\beta_{8}}$ , we obtain the impact of each independent variable on the use of smart integrated devices by the elderly.

To address potential multicollinearity issues and improve variable selection, Lasso (Least Absolute Shrinkage and Selection Operator) regression was employed. Lasso is a statistical method for regression analysis and feature selection, introduced by Tibshirani, 1996. By incorporating an L1 regularization term into the regression model, Lasso not only ensures better model fitting but also shrinks some regression coefficients to zero, thereby performing automatic variable selection. The objective function of Lasso is:


$$\min\sum_{i=1}^{n}{\left(y_{i}-\beta_{0}-\sum_{j=1}^{p}\beta_{j}x_{\mathrm{ij}}\right)}^{2}+\lambda \sum_{j=1}^{p}|\beta_{j}|$$

where is a tuning parameter that controls the degree of shrinkage. This method is particularly useful for identifying the most relevant factors influencing the use of smart integrated devices while preventing overfitting. By integrating logistic regression and Lasso analysis, this study ensures a robust and reliable examination of the determinants of digital adoption among older adults, providing a strong empirical basis for policy recommendations.

## 4 Results

Based on the results of the multilevel logistic regression model reported in Table 2, we can derive research conclusions regarding the factors influencing smart device use among the elderly.

**Table 2:** Multilevel logistic regression modeling of factors influencing smart device use among Chinese older adults.

Variables	(1)	(2)	(3)	(4)
	m1	m2	m3	m4
	smart_device	smart_device	smart_device	smart_device
Gender	0.244**	0.215*	0.200	0.213*
	(0.123)	(0.125)	(0.125)	(0.128)
Age	−0.0442***	−0.0316***	−0.0203*	0.0272**
	(0.0109)	(0.0111)	(0.0122)	(0.0136)
Living_status	0.478***	0.437**	0.434**	0.347*
	(0.178)	(0.180)	(0.179)	(0.185)
Health	−0.0953	−0.182	−0.187	−0.239*
	(0.127)	(0.130)	(0.130)	(0.134)
Education		0.300***	0.277***	0.194***
		(0.0668)	(0.0678)	(0.0697)
Income		−1.64e-05***	−1.60e-05***	−1.37e-05**
		(5.86e-06)	(5.85e-06)	(5.80e-06)
Child			−0.188**	−0.179**
			(0.0843)	(0.0852)
Internet				1.475***
				(0.168)
Constant	0.844	−1.081	−1.447	−5.412***
	(0.822)	(0.942)	(0.955)	(1.082)
Hosmer-Lemeshow χ^2^	12.34 (*p* = 0.14)	10.76 (*p* = 0.21)	10.49 (*p* = 0.23)	8.69 (*p* = 0.37)
Cox-Snell *R*^2^	0.123	0.145	0.158	0.197
Nagelkerke *R*^2^	0.165	0.194	0.213	0.267
Model χ^2^	45.67***	56.39***	64.58***	78.92***
Observations	2,330	2,330	2,330	2,330

Standard errors in parentheses. ***p < 0.01, **p < 0.05, *p < 0.1.

Firstly, gender shows a significant positive correlation with the use of smart devices among the elderly (odds ratio= $e^{0.213}$ ). Elderly males are more likely to seek the use of smart devices, which may be attributed to their higher enthusiasm for adopting and using technology. Age is also significantly positively correlated with the use of smart devices. As the age of elderly individuals increases, the probability of using smart devices also significantly increases (odds ratio= $e^{0.0272}$ ). Living status is another factor that significantly influences the use of smart devices (odds ratio= $e^{0.347}$ ). Married elderly individuals are more likely to use smart devices, which could be due to the support and encouragement from their spouses. Education level has a very significant impact on the use of smart devices (odds ratio= $e^{0.194}$ ). The higher the educational attainment, the more likely the elderly are to use smart devices. This indicates that individuals with higher education levels are more exposed to and accepting of new technologies, using them widely in their daily lives. Health status is significantly negatively correlated with the use of smart devices among the elderly (odds ratio= $e^{-0.239}$ ). This suggests that elderly individuals with poorer health are more likely to seek the use of smart integrated devices, possibly due to their need for health monitoring and assistance.

Income is also negatively correlated with the use of smart devices. This implies that elderly individuals with better economic status are less dependent on smart integrated devices, possibly because they have access to alternative resources or services. The number of children an elderly person has is negatively related to the use of smart integrated devices. The more children the elderly have, the lower the probability of using smart integrated devices. This might be because they receive more direct assistance from their children. Finally, internet usage experience positively affects the use of smart integrated devices among the elderly. Elderly individuals with prior internet usage experience are more likely to use smart integrated devices, as they are more familiar with digital technology and its benefits.

The results of the multilevel logistic regression analysis demonstrate that the models provide a good fit and explain a meaningful portion of the variance in smart device use among Chinese older adults. The Hosmer-Lemeshow test shows that the models fit the data well, with *p*-values ranging from 0.14 to 0.37, indicating no significant misfit. The Cox-Snell and Nagelkerke *R*^2^ values increase progressively across models, with Nagelkerke *R*^2^ reaching 0.267 in the final model, suggesting that the models explain a moderate proportion of the variance in smart device use. Additionally, the Model Chi-Square statistics are highly significant (*p* < 0.001) for all models, confirming that the predictors collectively contribute to the explanation of smart device use. These results support the robustness of the findings and the validity of the models in explaining the factors influencing smart device use among older adults.

**Figure 1:**
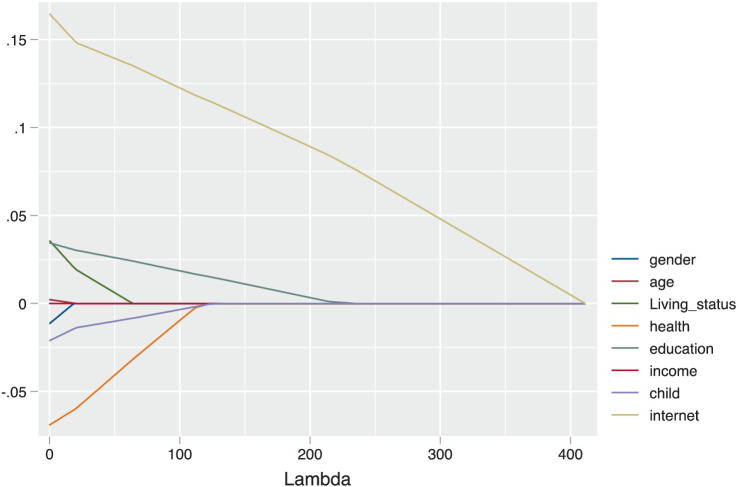
Factors most influencing the use of smart all-in-one devices by older adults in China (lasso model estimates).

Furthermore, to delve deeper into the relative importance of various factors influencing the use of smart integrated devices by the elderly, we employed the Lasso estimation method. Lasso estimation not only handles high-dimensional data but also effectively performs variable selection and regularization, thus avoiding multicollinearity issues. Our analysis revealed that the internet usage experience of the elderly is the most significant and enduring factor affecting their use of smart integrated devices. This factor demonstrated a substantial impact within our model, as shown in Figure 1. The findings indicate that elderly individuals with prior internet usage experience are more likely to adapt to and use smart integrated devices, providing a crucial basis for formulating policies to promote the digital inclusion of the elderly.

In the preceding sections, we identified internet usage experience as the most significant factor influencing the use of smart integrated devices among the elderly. Building on this finding, we further explored the impact of different internet usage purposes on the use of smart integrated devices by the elderly. To this end, we constructed interaction terms between internet usage and various activities, such as voice and video chatting, shopping, reading news, obtaining information, transportation, and financial investments (e.g., stock trading, buying funds). The results of our study are presented in Table 3.

**Table 3:** The impact of different internet usage uses on the use of smart all-in-one devices for the elderly in China.

Variables	(1)	(2)
	m1	m2
	smart_device	smart_device
Internet × chat	−1.211**	−1.143**
	(0.476)	(0.485)
Internet × shopping	−0.00668	−0.0118
	(0.213)	(0.217)
Internet × watching	−0.132	−0.0682
	(0.178)	(0.184)
Internet × getting information	0.768***	0.860***
	(0.185)	(0.191)
Internet × transport	−0.801***	−0.760***
	(0.214)	(0.217)
Internet × invest	−0.729**	−0.721**
	(0.292)	(0.301)
Control variables	No	Yes
Hosmer-Lemeshow χ^2^	8.45 (*p* = 0.39)	7.92 (*p* = 0.44)
Cox-Snell *R*^2^	0.127	0.151
Nagelkerke *R*^2^	0.169	0.202
Model χ^2^	48.75***	57.34***
Observations	1,251	1,251

Standard errors in parentheses. ***p < 0.01, **p < 0.05, *p < 0.1.

The study revealed that elderly individuals who rely more on the internet for voice and video chatting with family and friends tend to use smart integrated devices less frequently. This might be because their social needs are already being met through other means, reducing their dependence on smart integrated devices. Conversely, elderly individuals who primarily use the internet to obtain information may have poorer health and therefore rely more on smart integrated devices for accessing medical and health-related information and services.

Furthermore, the study showed that elderly individuals who use the internet mainly for transportation arrangements, indicating more outdoor activities, tend to be less dependent on smart integrated devices. In contrast, those who use the internet predominantly for financial investments focus more on the financial markets and thus have less need for the companionship provided by smart integrated devices.

The results suggest that the models fit the data well, as indicated by the Hosmer-Lemeshow test (*p* > 0.05 for both models). The explanatory power of the models is moderate, with the Nagelkerke *R*^2^ increasing from 0.169 in model m1 to 0.202 in model m2, reflecting a better explanation of the variance in smart device use when control variables are included. Both models are statistically significant, with model Chi-Square values of 48.75 for m1 and 57.34 for m2 (*p* < 0.001).

In summary, our research highlights the role of smart integrated devices in providing companionship to the elderly and underscores the varying impacts of different internet usage purposes on this companionship role. These findings offer valuable insights for further enhancing the user experience of smart devices among the elderly.

## 5 Discussion

This study provides a comprehensive analysis of the factors influencing the adoption of smart integrated devices among the elderly. The findings highlight key demographic, social, and technological determinants, emphasizing the role of prior internet usage experience, which emerged as the most significant predictor. The use of Lasso regression allowed for the systematic identification of the most relevant factors without overfitting the model, demonstrating a methodological advancement over previous studies.

The results indicate that gender plays a significant role in the adoption of smart devices among the elderly, with males showing a higher likelihood of using these devices. This can be attributed to several factors, including a generally higher enthusiasm for technology among older males. Men might also be more inclined to explore and utilize new technologies due to their historical and cultural exposure to technological advancements. Age is a positively correlated factor, meaning that as individuals grow older, their likelihood of using smart devices increases. This finding is particularly interesting as it contrasts with the common perception that younger elderly individuals might be more technologically adept. One explanation is that smart integrated devices, particularly voice assistants and health monitoring systems, serve as assistive tools for daily life rather than mere entertainment gadgets. Younger elderly individuals may not yet require these functionalities, whereas older seniors, who experience greater health challenges and mobility restrictions, are more likely to rely on such technologies for assistance and safety. This finding aligns with the notion that smart devices play a crucial role in supporting independent living among older adults, providing functionalities that compensate for declining physical and cognitive abilities.

Furthermore, the results indicate that internet usage experience is the most significant and enduring factor influencing smart device adoption among the elderly. Elderly individuals with prior exposure to the internet are more likely to embrace smart devices, as they are already accustomed to digital interactions and understand the benefits of these technologies. This underscores the importance of digital literacy programs, which can bridge the gap for those with limited prior exposure and facilitate broader technology adoption among aging populations.

Income level exhibited a somewhat unexpected trend, revealing that lower-income elderly individuals are more likely to use smart devices. This result challenges the assumption that smart technologies are primarily accessible to wealthier individuals. In China, the affordability of smart home products and digital devices makes them widely accessible, even to lower-income groups. Compared to other countries where such devices may be considered luxury items, the relatively low cost of consumer electronics in China promotes widespread adoption across different economic demographics. This highlights the inclusivity of digital solutions and their potential to support elderly individuals across various socioeconomic backgrounds. Another possible explanation is that low-income elderly individuals may rely more on smart devices for social support and daily life assistance compared to higher-income groups. Due to limited financial resources, they may not have access to expensive caregiving services or offline social interactions, making them more inclined to use smart devices for information access, social engagement, health management, and government benefits. For example, smart voice assistants, online medical consultations, and digital services provided by the government can compensate for the lack of real-life resources, enabling them to better adapt to modern society. Additionally, smart devices offer cost-effective entertainment and communication options, helping to reduce social isolation, which could be another reason why low-income groups depend more on these technologies.

Another key finding relates to marital status and family structure. Married elderly individuals are more likely to use smart devices, likely due to encouragement and support from their spouses. In contrast, elderly individuals with more children tend to rely less on digital technology, as they receive in-person care and support from family members. This suggests that family networks play a dual role: they can either facilitate or substitute the need for technology-based assistance. Policymakers should consider these nuances when designing interventions to promote digital inclusion among older populations.

## 6 Conclusion and policy implications

In This study stands out by focusing on the urban elderly population in China and employing a nationally representative dataset combined with advanced analytical methods. By incorporating Lasso regression, this study identifies the most critical determinants influencing smart device adoption among elderly individuals, providing a methodological advantage over previous research. The use of Lasso allows for precise variable selection and minimizes multicollinearity issues, enhancing the robustness of the findings. These methodological innovations, along with the study’s focus on a specific population, contribute to a more comprehensive understanding of digital inclusion among the elderly.

In summary, this research highlights the multifaceted role of smart integrated devices in the lives of elderly individuals. Different factors, including gender, age, marital status, education level, health status, income, number of children, and internet usage experience, influence the adoption and use of these technologies. Additionally, the specific purposes of internet usage play a crucial role in determining the extent of reliance on smart devices. These findings offer valuable insights for policymakers and practitioners aiming to enhance digital inclusion and improve the user experience of smart devices among the elderly. By addressing the identified factors and tailoring interventions to the specific needs and contexts of seniors, broader and more effective adoption of smart technologies can be promoted in this demographic.

To better assist the elderly in sharing the benefits of the digital age and achieving the goal of positively addressing population aging, a concerted effort from families, society, and the government is required. Governments and communities should organize digital skills training targeted at the elderly, enhancing their acceptance and usage capabilities of smart devices. For elderly individuals with limited economic means, government subsidies and preferential policies can lower the barriers to using smart devices. Manufacturers of smart devices should focus on the needs of elderly users, designing devices that are simple to operate and practical in function. Family members and community workers should actively assist and support the elderly in using smart devices, offering necessary technical and psychological support. Through media publicity and policy promotion, increasing the elderly’s awareness and acceptance of smart devices can create a favorable social atmosphere.

By making concerted efforts across multiple domains, we can jointly create an elderly-friendly digital device environment. This can effectively promote the elderly’s adaptation to and integration into the digital society, enhancing their quality of life and well-being, and advancing sustainable societal development.

## 7 Limitations and future research

While this study provides meaningful insights, certain limitations must be acknowledged. First, the analysis is based on national survey data, which, while comprehensive, does not capture the qualitative dimensions of technology adoption among the elderly. Future research should incorporate qualitative methods, such as focus groups and in-depth interviews, to gain richer insights into elderly users’ experiences, challenges, and motivations.

Second, the findings are based on cross-sectional data, which limits the ability to infer causal relationships. A longitudinal study would help track changes in smart device adoption patterns over time, providing stronger evidence of causality between influencing factors and adoption behaviors.

Third, cultural and social factors, such as familial caregiving expectations and social stigmas surrounding technology use in older age, were not explicitly analyzed. Future research should explore how cultural dynamics influence digital inclusion among the elderly, particularly in different geographic and economic contexts.

By addressing these limitations in future studies, researchers can further refine the understanding of digital adoption among the elderly and contribute to the development of more effective interventions that promote digital inclusion in aging societies.

## Data Availability

This study is based on the publicly available dataset from the 2020 China Longitudinal Aging Social Survey (CLASS). Researchers interested in accessing the data may obtain it through public search or by contacting the corresponding author.
